# Supplementary material for: Poly(ADP)-Ribosylation Inhibition: A Promising Approach for Clear Cell Renal Cell Carcinoma Therapy
Source: Cancers (Basel). 2021 Oct 3;13(19):4973. doi: 10.3390/cancers13194973 (PMC8507656; doi:10.3390/cancers13194973)
Supplement: Supplementary file 1 [file cancers-13-04973-s001.zip › cancers-1338923-SI.pdf]

Supplementary Materials: Poly(ADP)-Ribosylation Inhibition: A Promising Approach for Clear Cell Renal Cell Carcinoma Therapy

Yaroslava Karpova, Danping Guo, Peter Makhov, Adam M. Haines, Dmitriy A. Markov, Vladimir Kolenko and Alexei V. Tulin

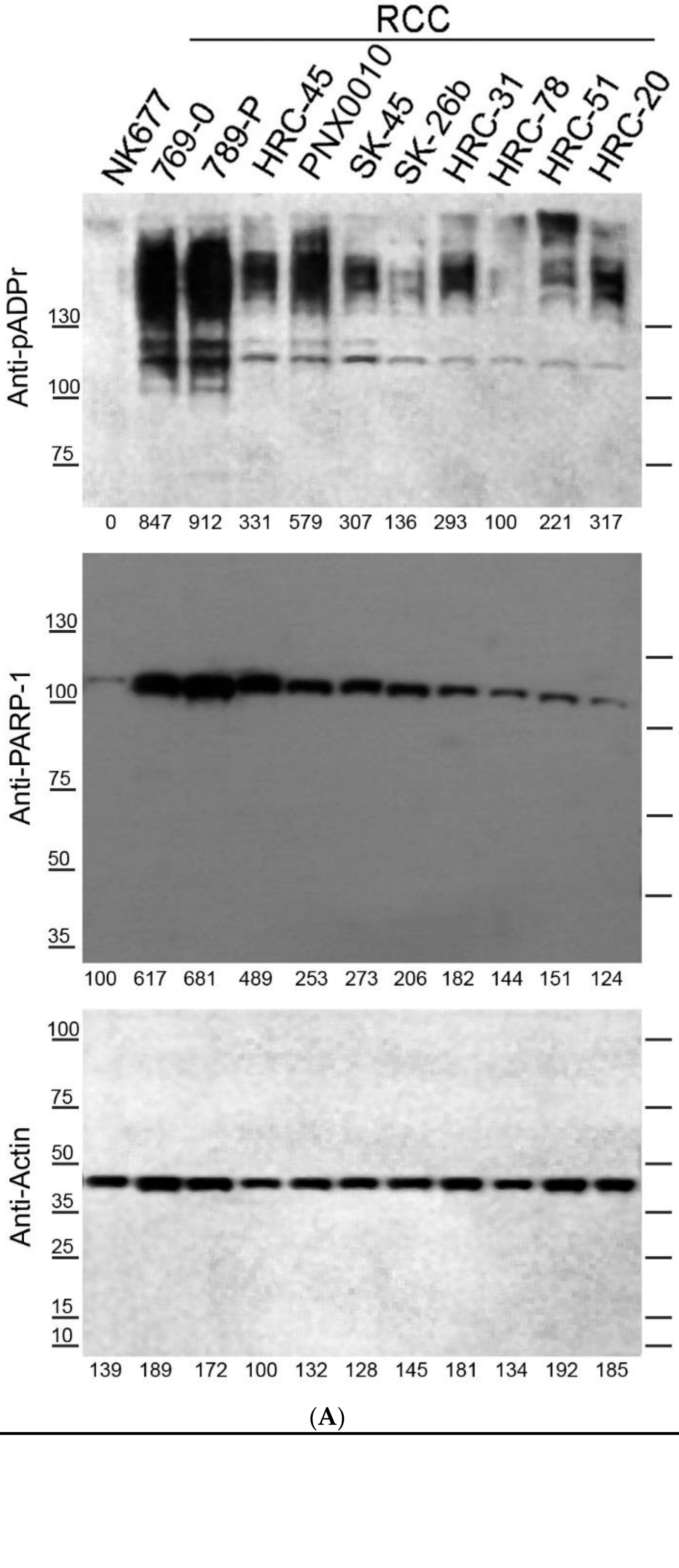

(**A**)

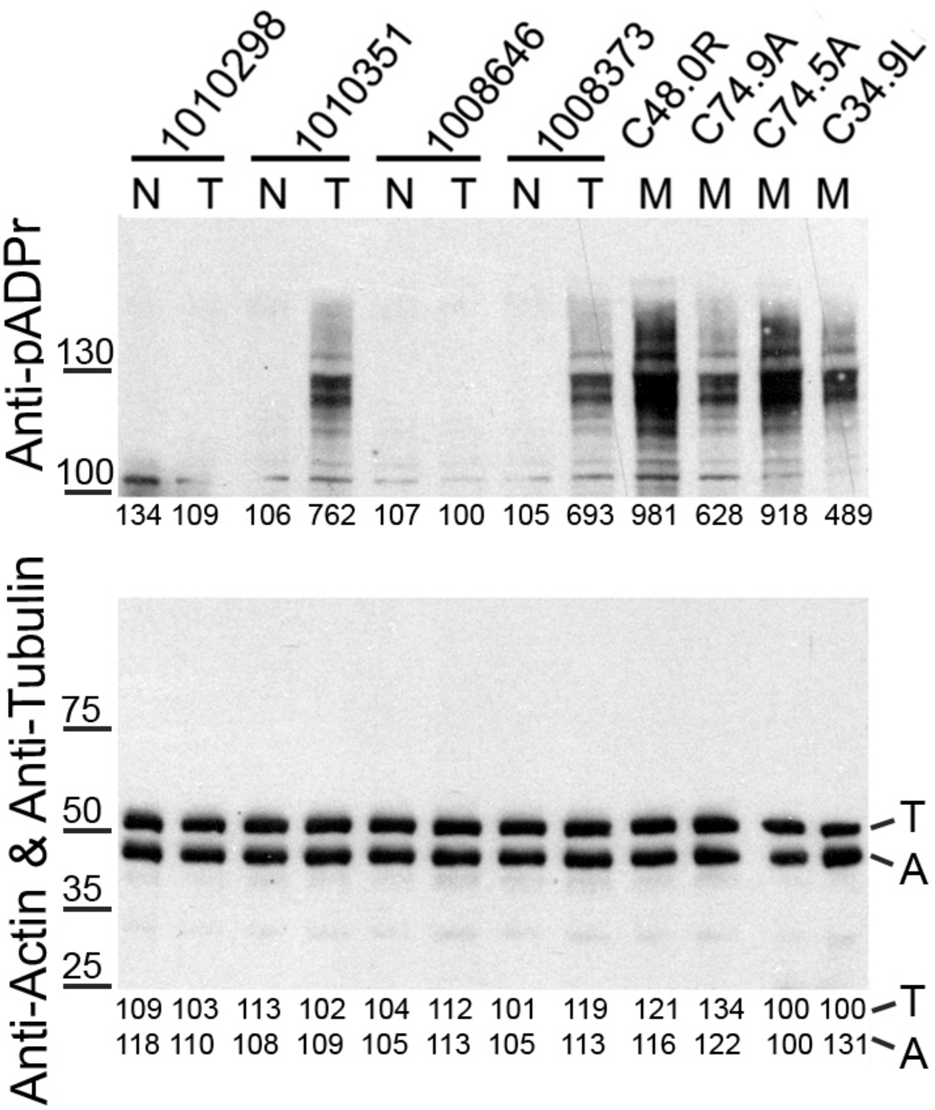

(**B**)

Figure S1. ccRCC cell lines and tumors affected by pADPr turnover. Western blot analysis for pADPr and PARP-1 in ccRCC cell lines (**A**) and patient-derived tumors (**B**). "N", "T", and "M" indicate "normal", "tumor" and "metastatic" samples, respectively. All studied ccRCC samples demonstrate a high level of pADPr. Actin and Tubulin levels are shown as a loading control.

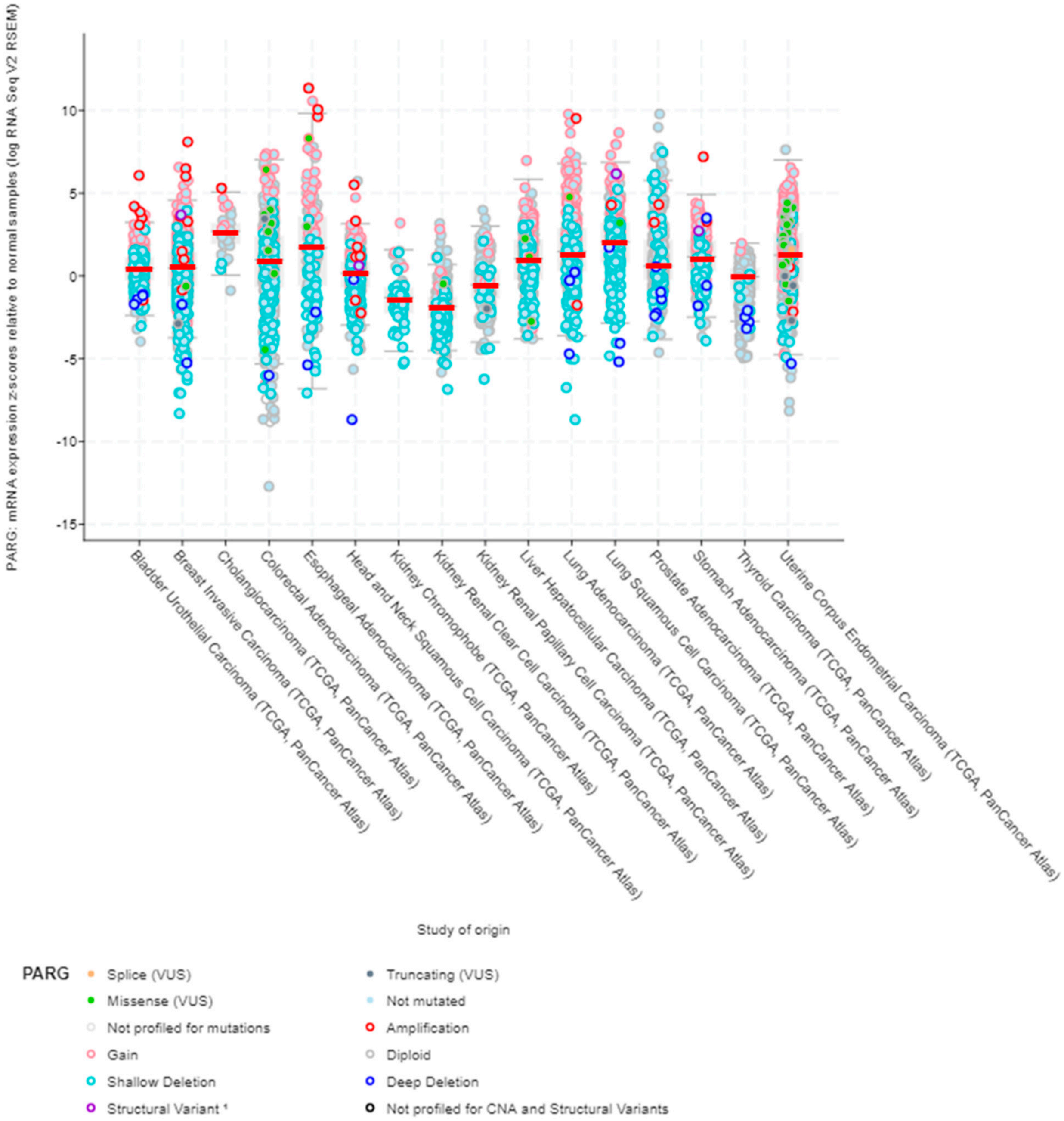

Figure S2. ccRCC (red box) exhibit the most sever downregulation of mRNA expression in tumor relative to normal samples. Graph generated at cBioPortal (https://www.cbioportal.org/) for TCGA PanCancer Atlas RNA sequencing data for different types of cancer and corresponding normal tissue.

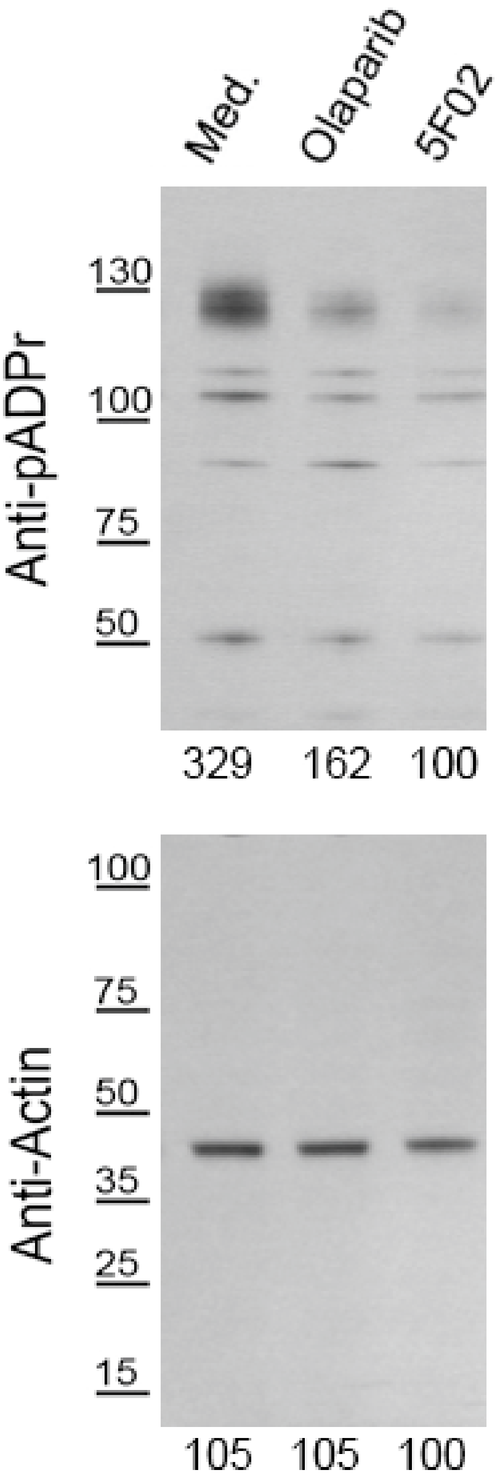

Figure S3. Western blot analysis shows the reduction of pADPr level in tumors upon olaparib and 5F02 PARPs inhibition. Actin level is shown as loading control.

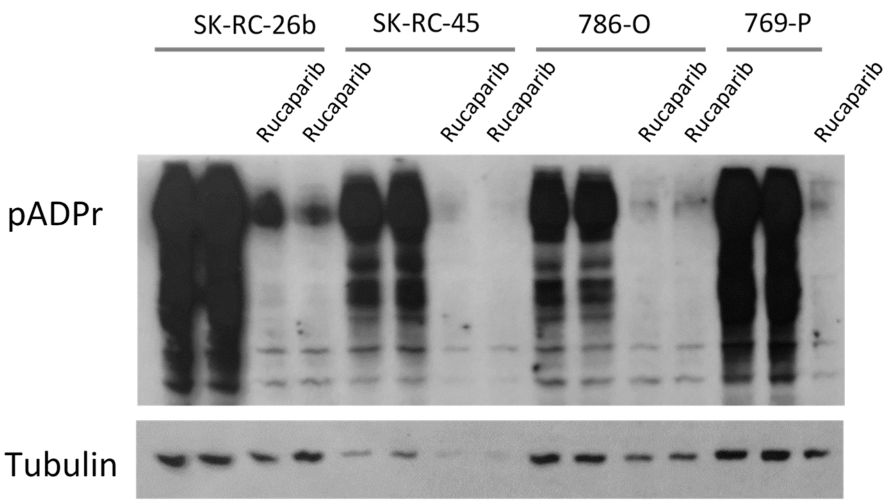

Figure S4. pADPr inhibition in ccRCC cell lines treated with 10 uM rucaparib for 48 h. Tubulin level is shown as a loading control.

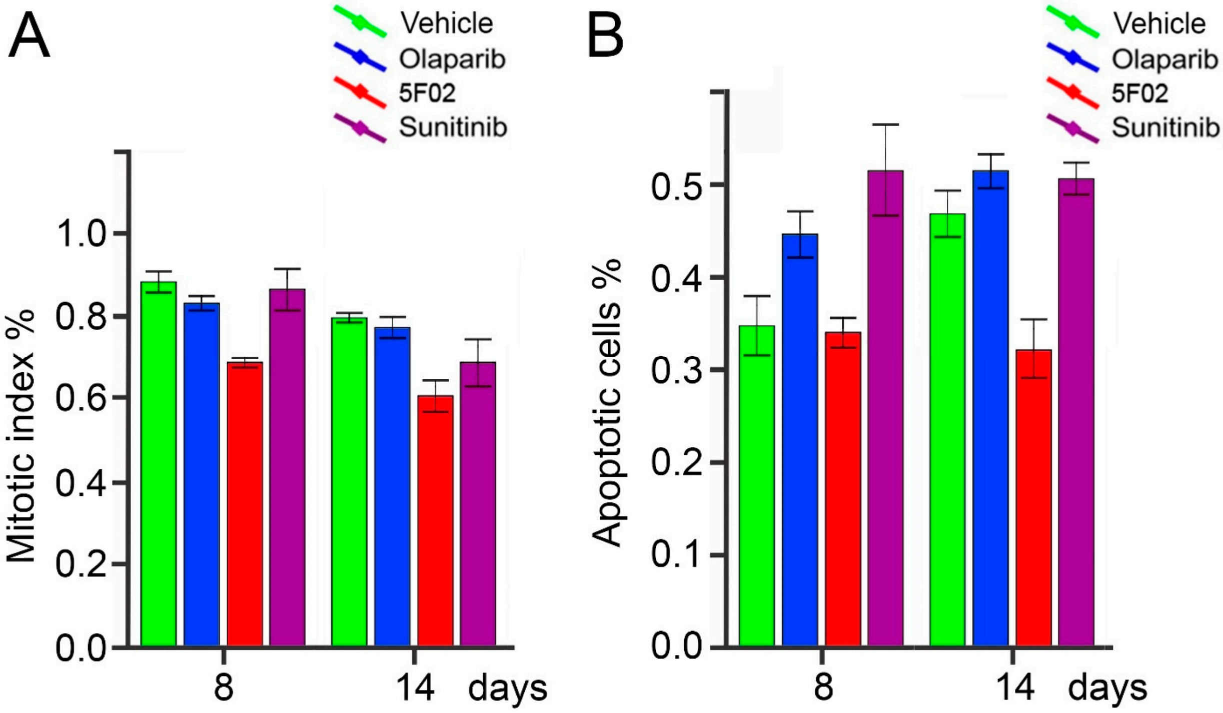

Figure S5. Proliferation index (**A**) and apoptosis level (**B**) in PNX0010 derived xenograft tumors 8 days and 14 days after tumor induction. Error bars correspond to standard deviation based on 8 repeats.

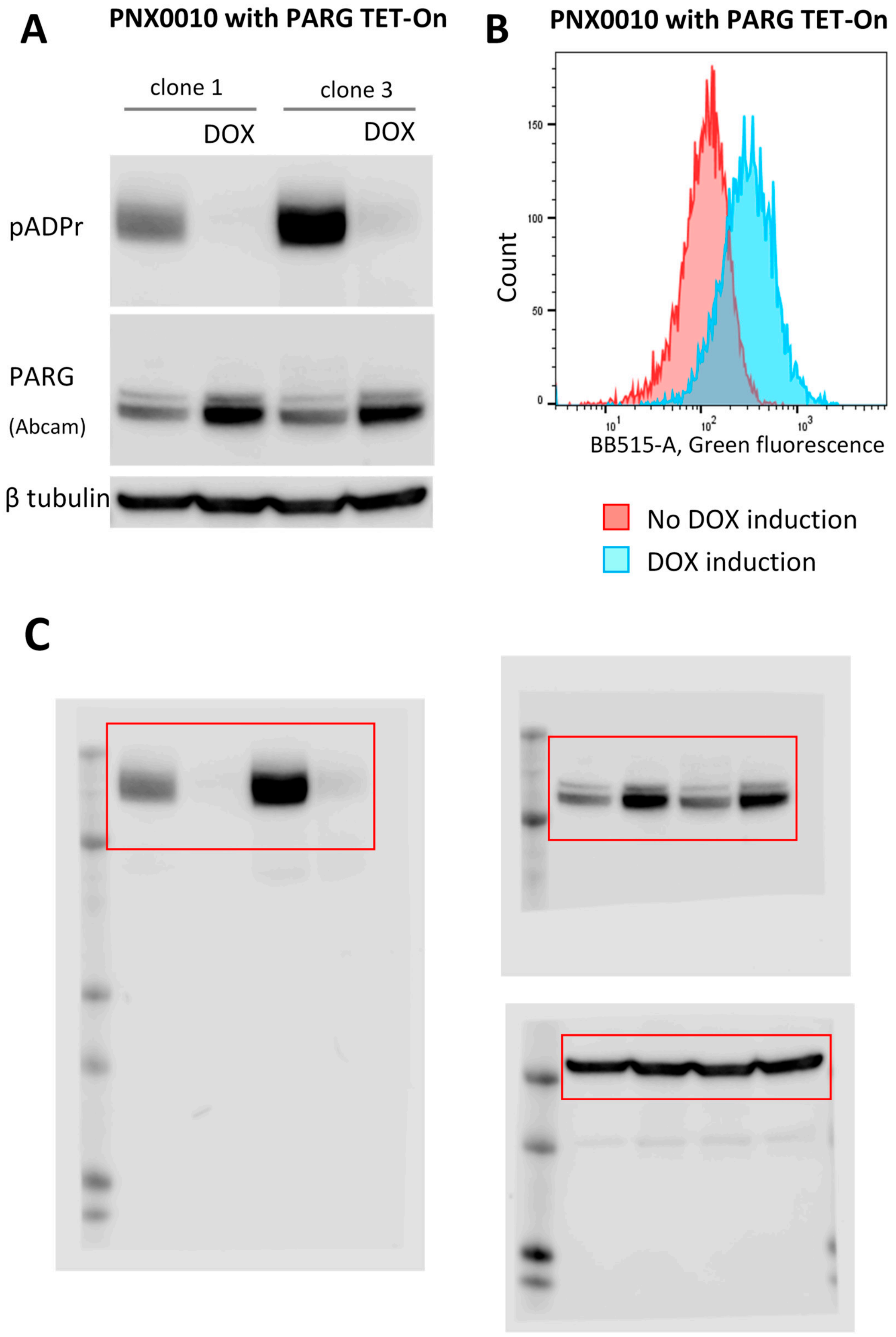

Figure S6. PARG overexpression and pADPr reduction in transduced PNX0010 cells**.** (**A**) pADPr inhibition in PNX0010 cell lines overexpressing PARG under 500 ng/ul doxycycline stimulation for 72 h. Two clones #1 and #3 are shown. Tubulin level is shown as a loading control. (**B**) Flow cytometry of live PNX0010 cell line with PARG TET-On lentovirus construct without doxycycline induction (red) and with doxycycline induction (blue). Because cDNA PARG is followed by cleavable mClover3 tag, cell numbers with increased green fluorescence is noticeable on histogram under doxycycline treatment. (C) Uncropped versions of Western Blots from (A).

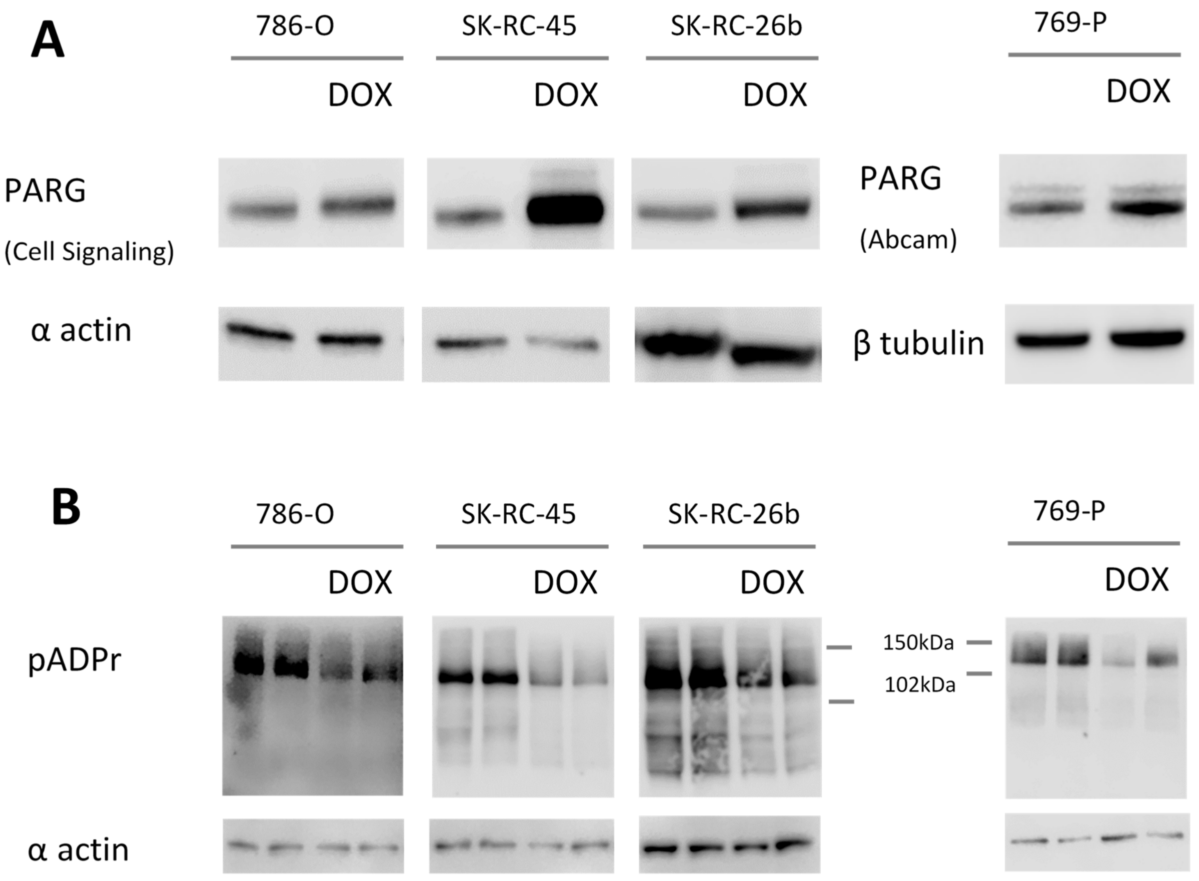

Figure S7. PARG overexpression and pADPr reduction in transduced ccRCC cells. (A) PARG overexpression and (**B**) pADPr reduction in 786-O, 769-P, SK-RC-45, SK-RC-26b transduced with Lenti-X construct under 500 ng/ul doxycycline stimulation for 72 h. Western blotting was probed for PARG using antibody (Abcam or Cell Signaling as indicated on figure) and for pADPr using MABE1031 reagent (Millipore). β tubulin and α actin levels are shown as a loading control.

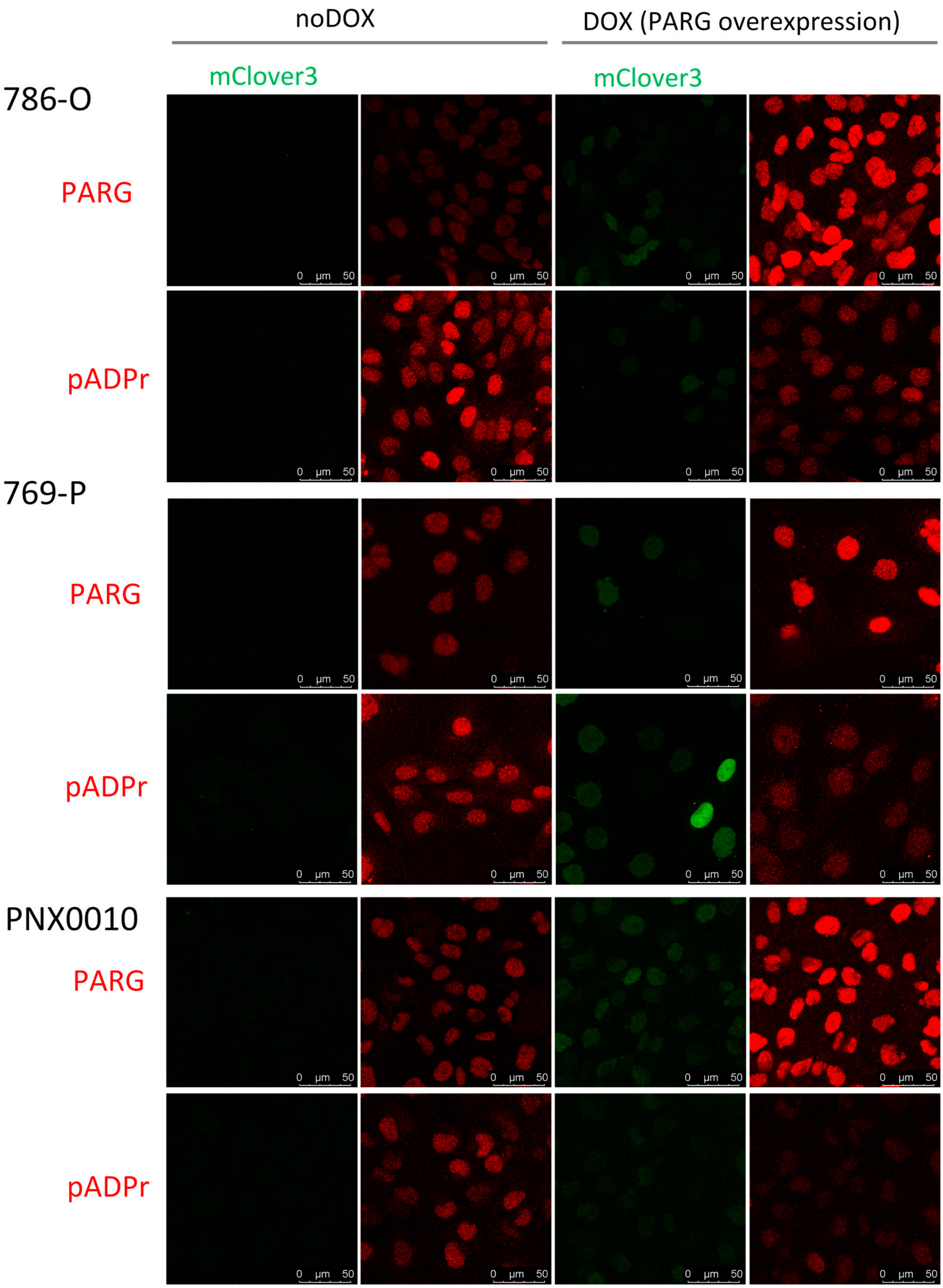
noDOX
DOX (PARG overexpression)
mClover3
mClover3
786-O
PARG
pADPr
769-P
PARG
pADPr
PNX0010
PARG
pADPr
0 µm 50

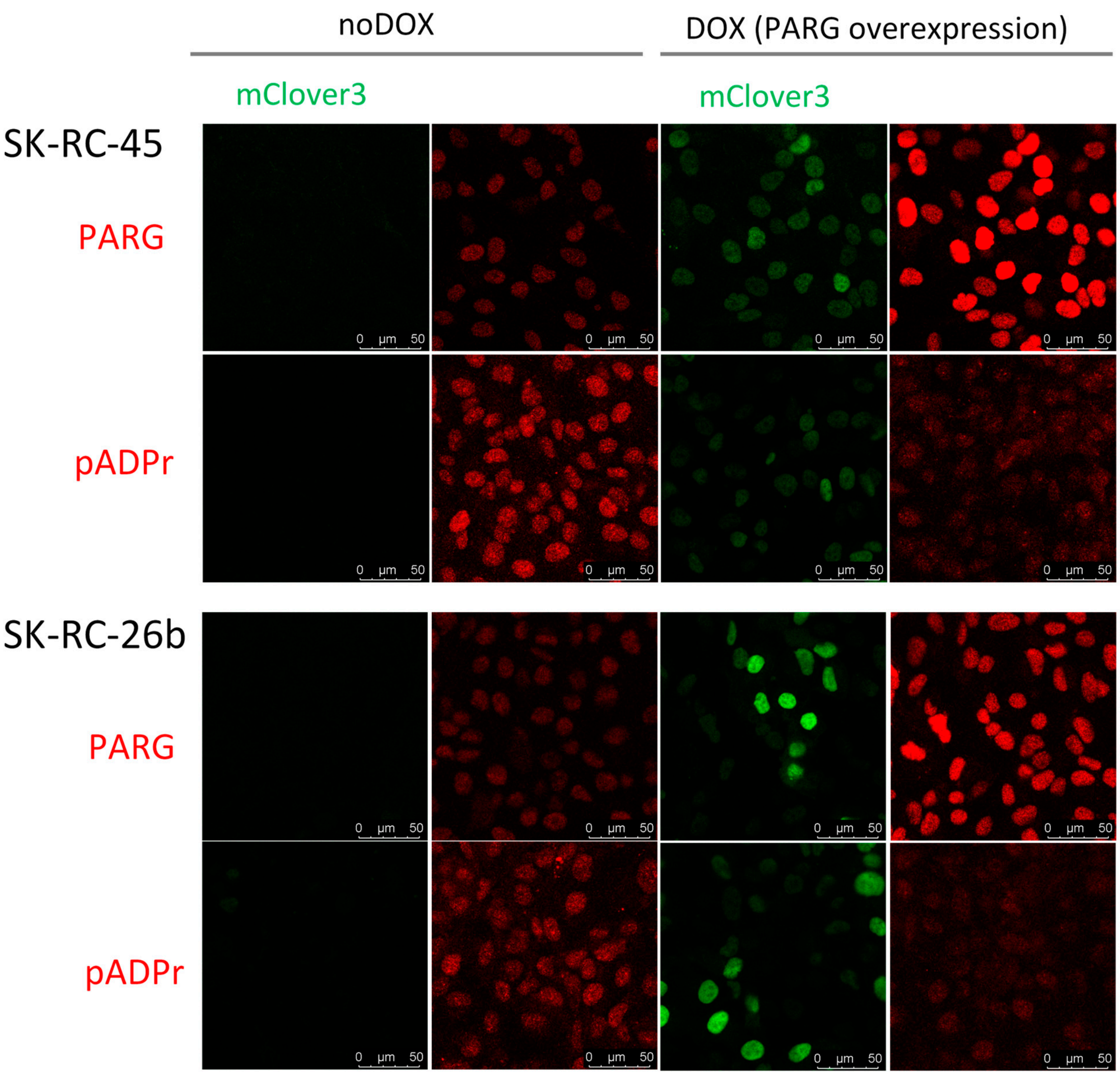

Figure S8. PARG overexpression and pADPr reduction in 786-O, 769-P, SK-RC-45, SK-RC-26b and PNX0010 transduced with PARG Tet-On Lenti-X construct under 500 ng/ul doxycycline stimulation for 72 h. Cells were fixed and stained for PARG using antibody (Cell Signaling) or for pADPr using MABE1031 reagent (Millipore) and corresponding Alexa 568 tagged secondary antibody. Green signal is from endogenous mCLover3 cleavable fluorescence tag.

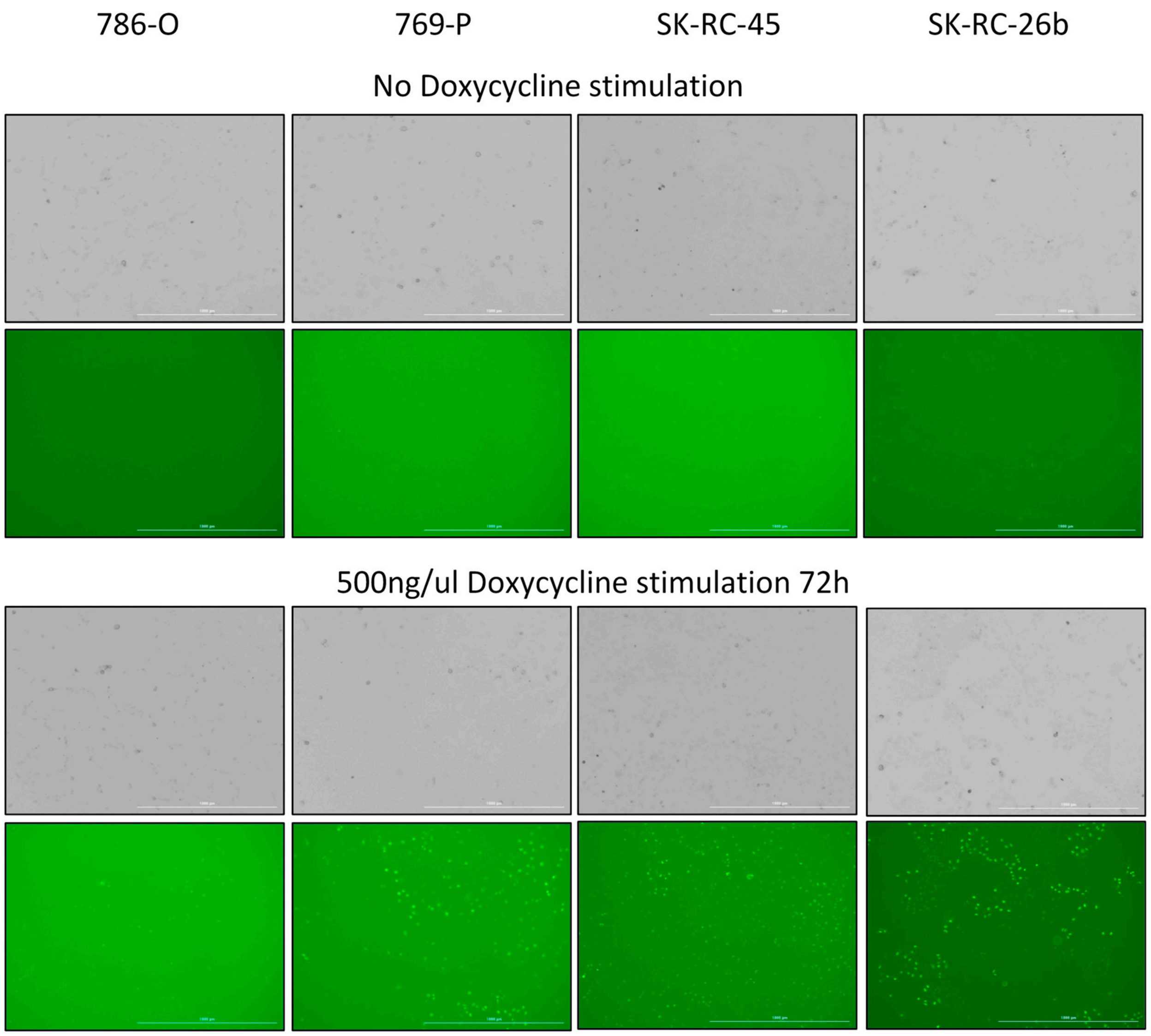

Figure S9. ccRCC cell lines were permanently transduced with lentivirus construct containing PARG cDNA followed by cleavable mCLover3 green fluorescence protein with NLS signal under TET-On doxycycline inducible promoter. Live imaging is present with green fluorescence visible after doxycycline induction of lentivirus containing ccRCC cells. White scale 1000 µm.

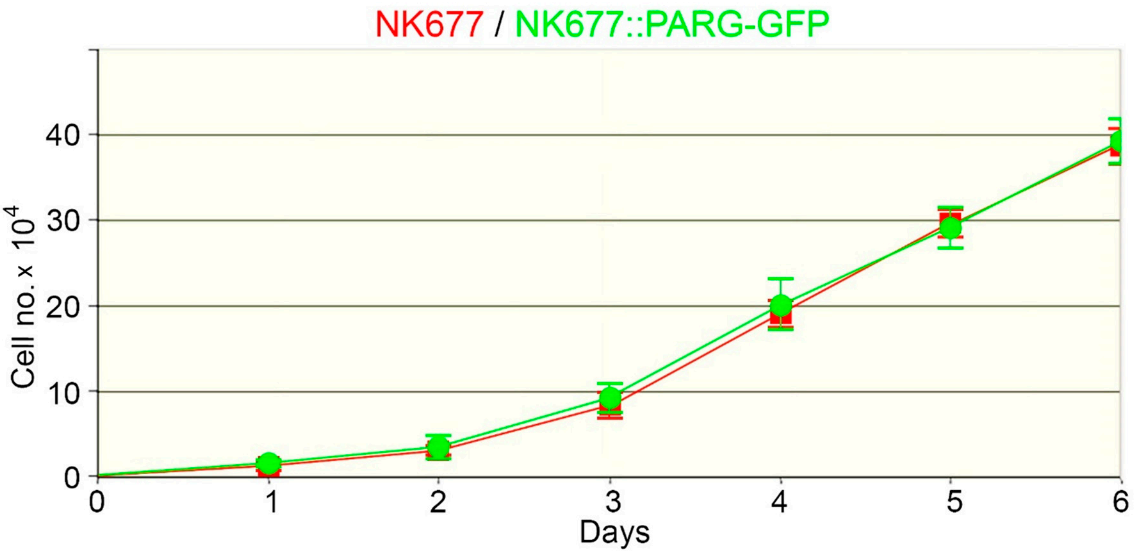

Figure S10. PARG protein overexpression does not affect the viability and the proliferation rate of normal kidney cells NK677.

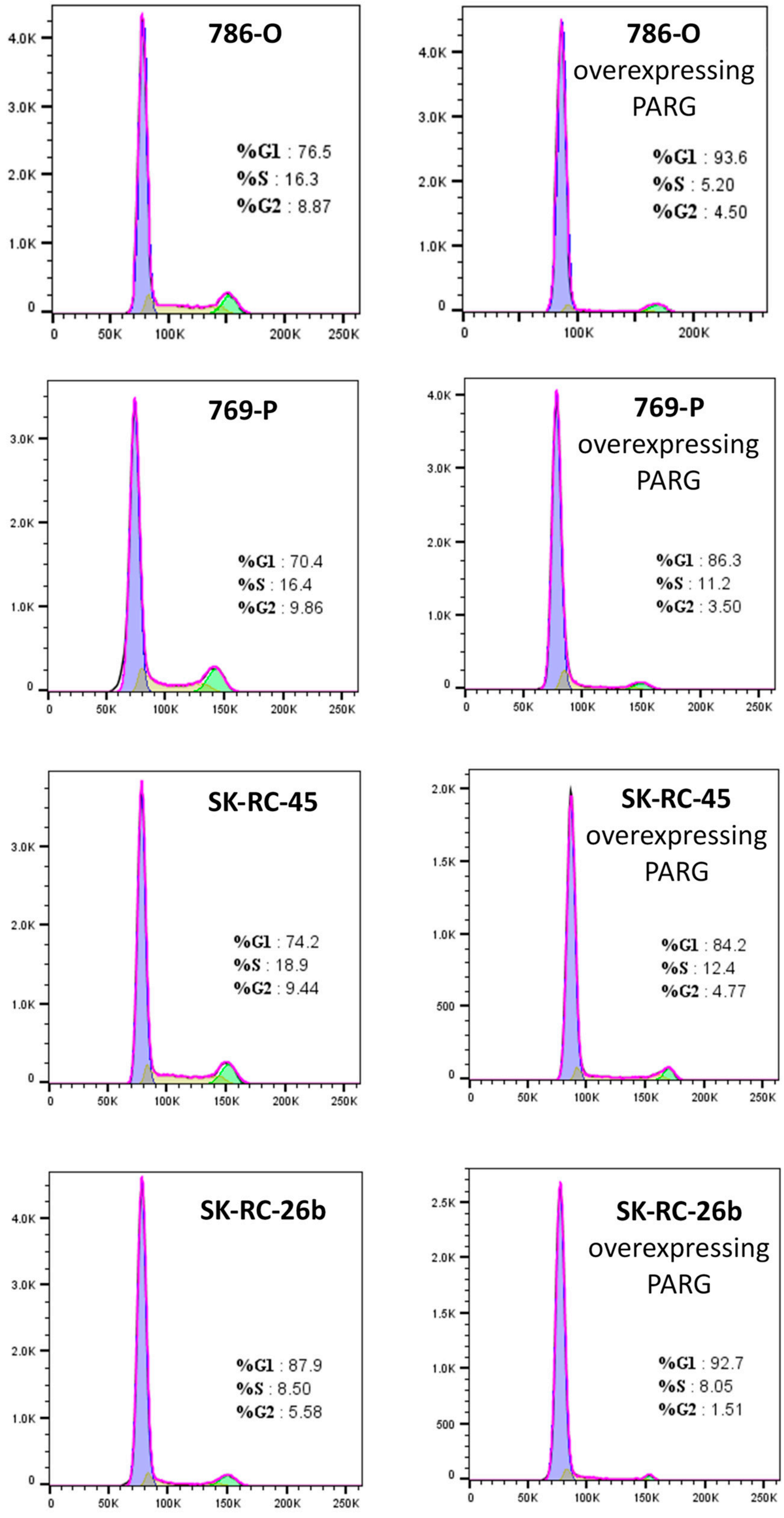

Figure S11. Histogram of cell cycle analysis ccRCC cells transduced with PARG Tet-On Lenti-X construct under 500 ng/ul doxycycline stimulation for 72 h.

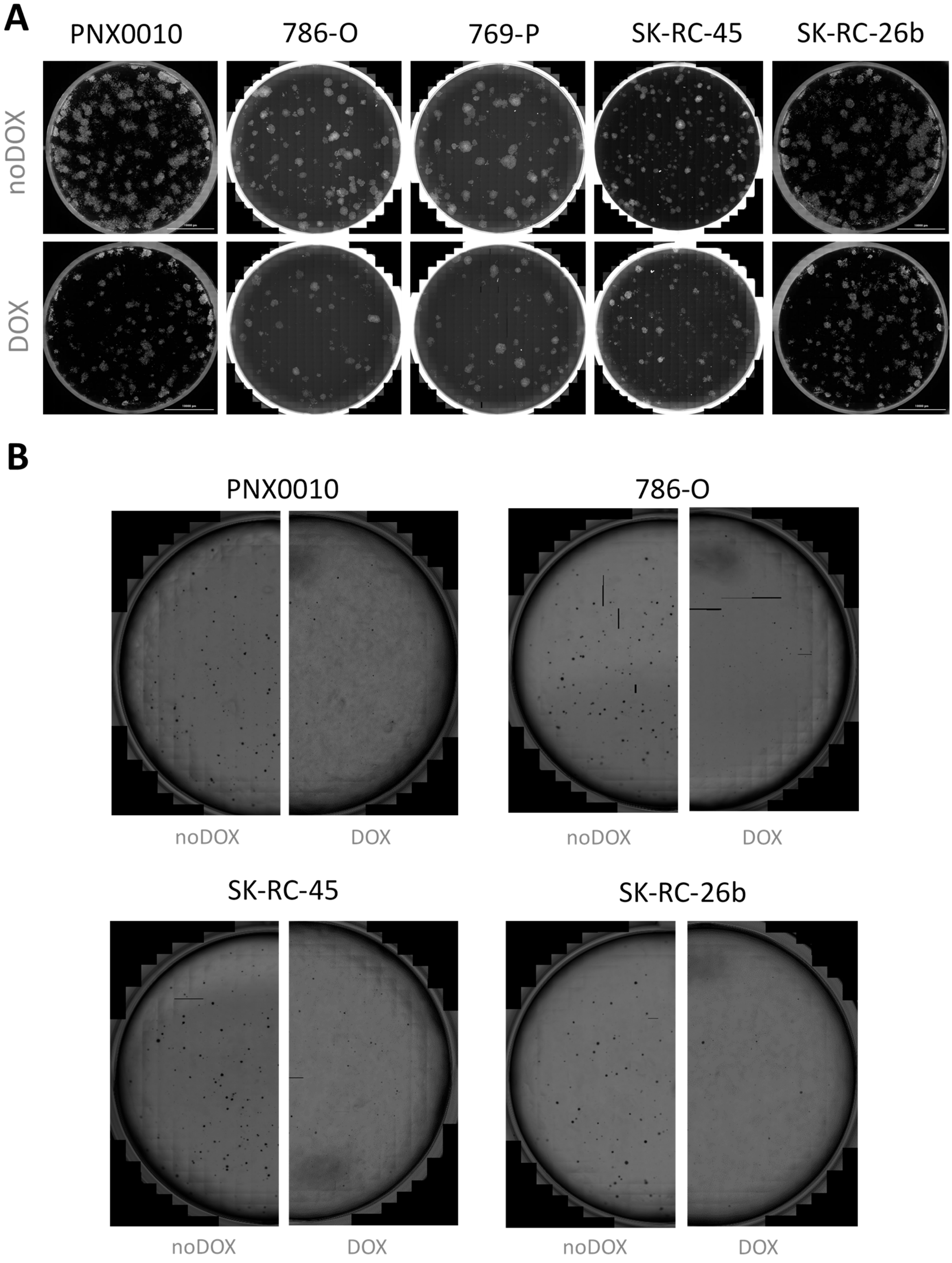

Figure S12. Images of ccRCC cells colonies transduced with PARG Tet-On Lenti-X construct with/without 500 ng/ul doxycycline stimulation for 72 h. Clonogenic (**A**) and colony-formation (**B**) assays are presented.

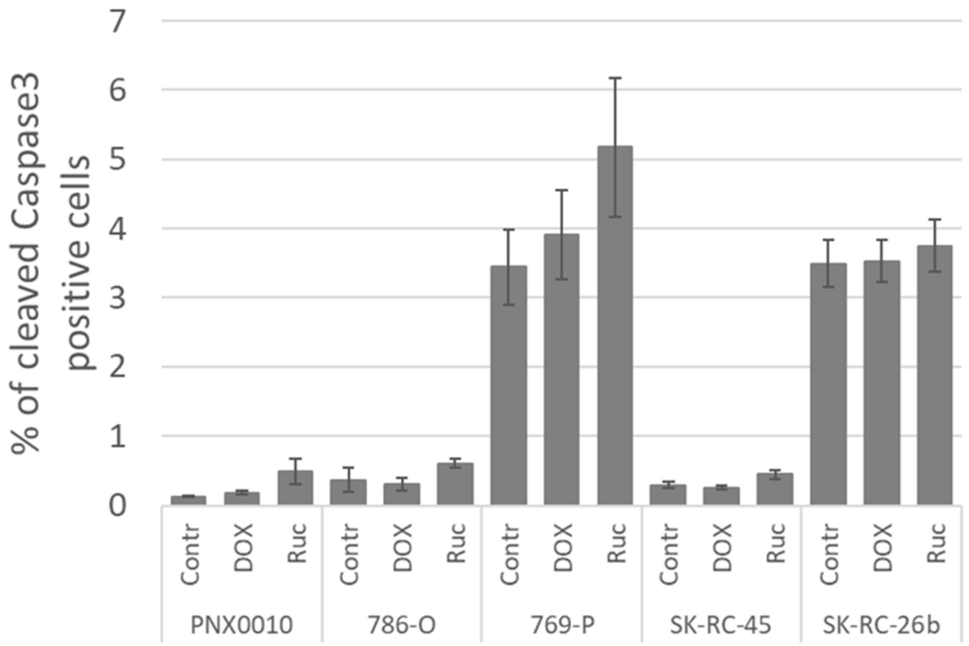

Figure S13. Apoptosis is not the cause of PARPs chemical inhibition and PARG overexpression related phenotypes in ccRCC cells. ccRCC cells transduced with PARG Tet-On Lenti-X construct were plated in 6 well dish in triplicates for each treatment groups: control with DMSO only, PARG overexpression with 500 ng/mL doxycycline for 72 hours and PARPs inhibition for 7.5 uM rucaparib for 48 hours. Cells were fixed and stained with antibodies to the marker of apoptotic cells cleaved Caspase3 and subjected to flow cytometry. % of cleaved Caspase3 positive cells were calculated in FlowJo software and plotted. We didn't detect significant increase of apoptotic cells between all treatment groups for all studied cell lines.

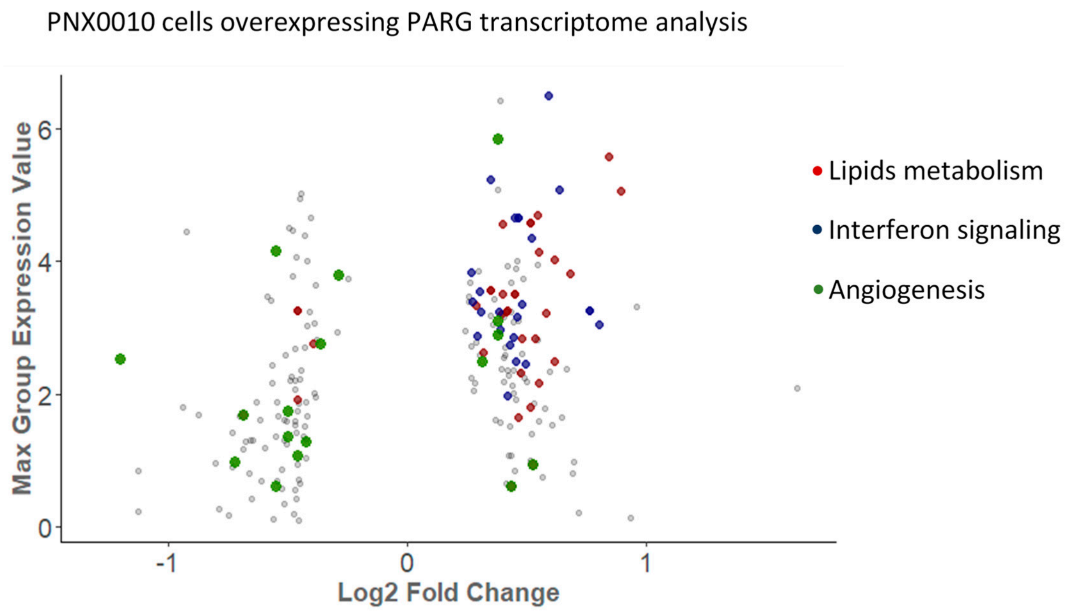

Figure S14. Maximum group expression value/Log2 fold change values for PNX0010 cells overexpressing PARG genes that differ with FDR corrected p value < 0.05 and maximum group expression value >1. Genes involved in Lipids metabolism (red) and Interferon signaling (blue) pathways are mostly found among upregulated genes and genes involved in Angiogenesis regulation (green) pathway are mostly found among downregulated genes colored red, blue and green respectively. Downregulated genes are enriched in angiogenesis regulation pathway, upregulated genes are enriched in cholesterol/lipids metabolism and interferon response.

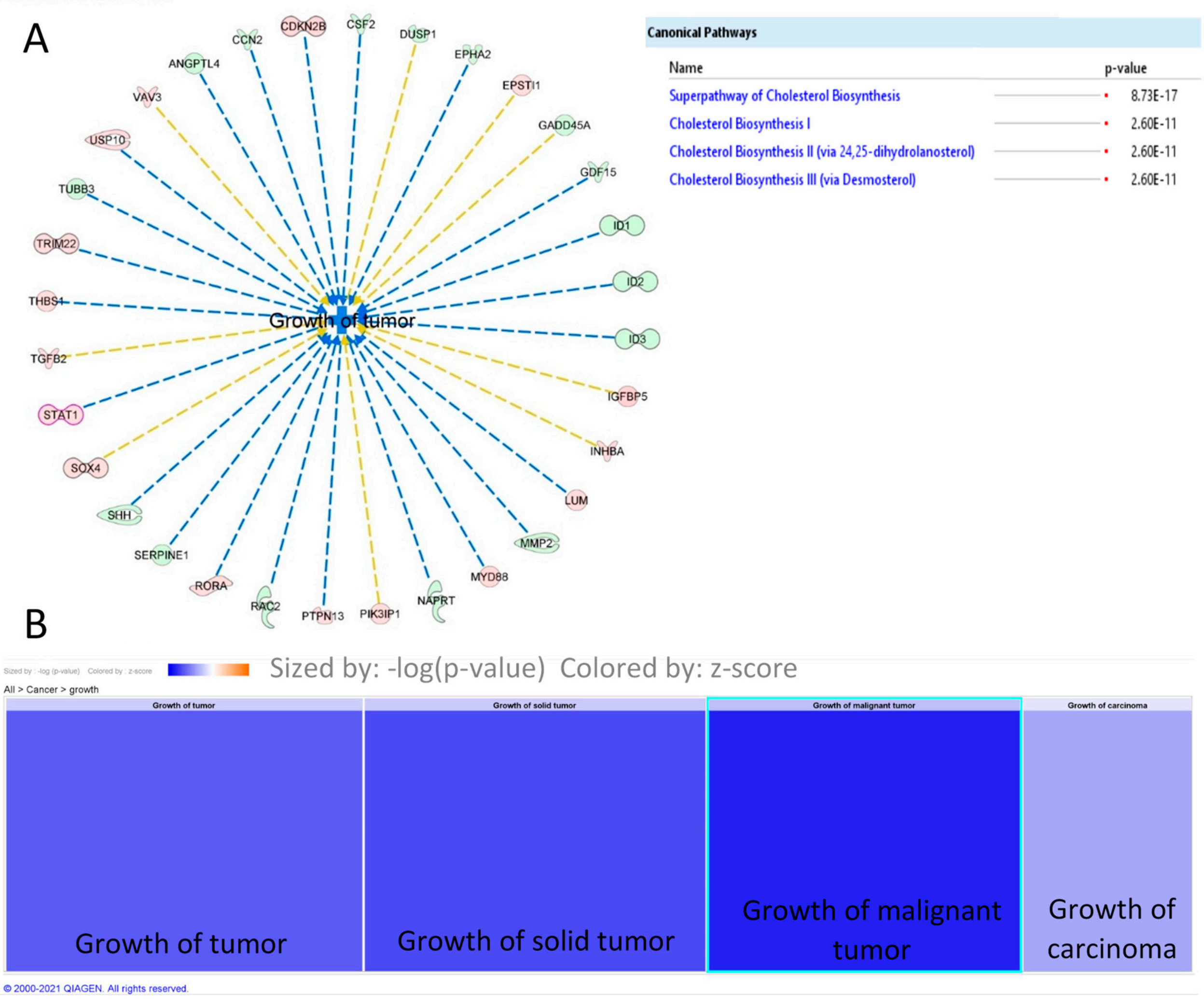

Figure S15. (**A**) Differently expressed genes in PNX0010 cells overexpressing PARG with FDR corrected *p* value < 0.05 and maximum group expression value > 1 involved in Growth of tumor disease phenotype predicted with Ingenuity Pathway Analysis software. (**B**) The overall phenotype is predicted to be downregulated.

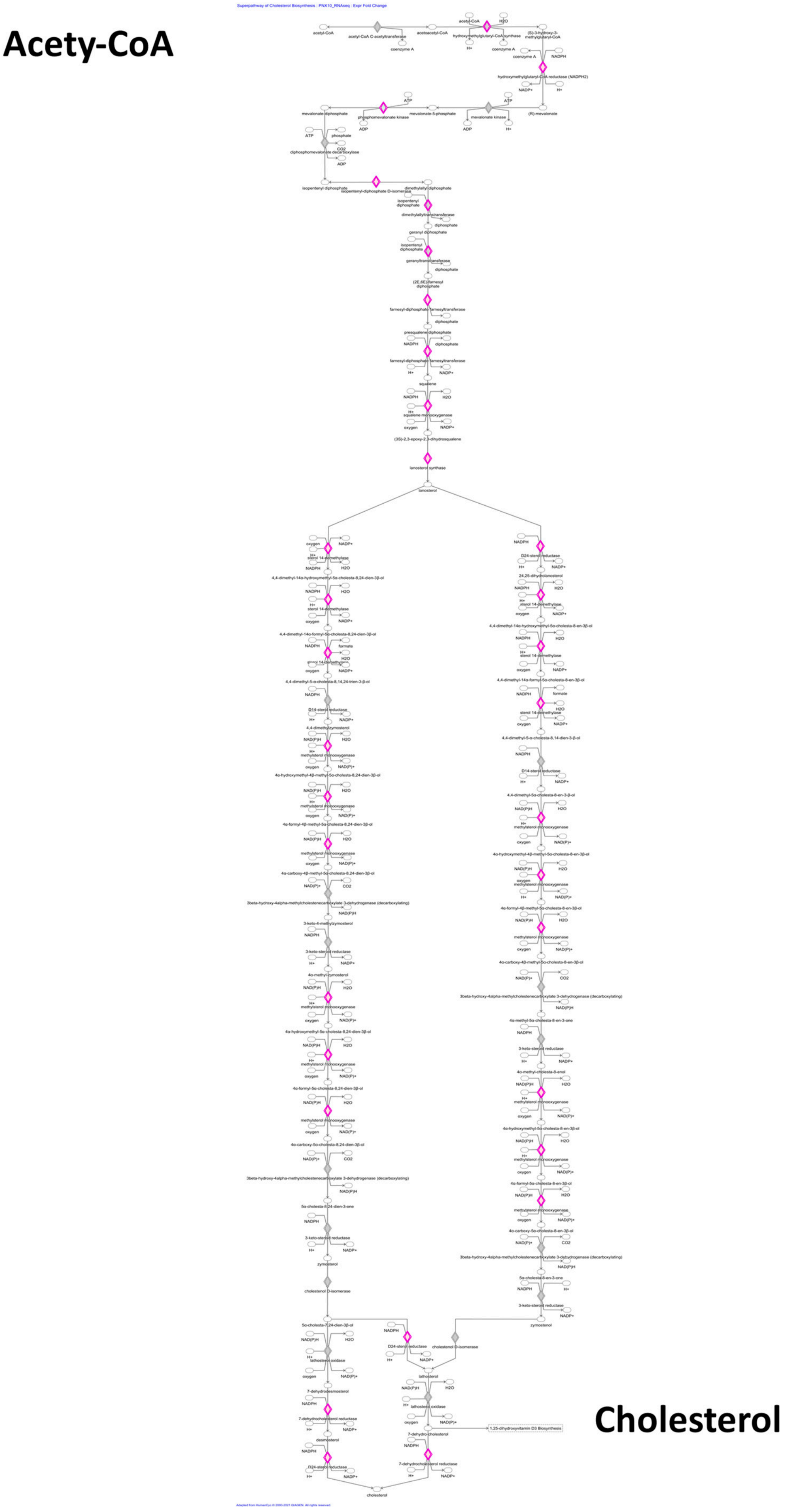
Acety-CoA
Cholesterol

Figure S16. Ingenuity Pathway Analysis software generated pathway of cholesterol biosynthesis with enzymes presented as diamonds and differently expressed in PNX0010 cells overexpressing PARG (FDR corrected *p* value < 0.05 and maximum expression value >1) colored as purple diamonds.

Downregulated genes with FDR <0.05, max group expression value >1.

	Homo sapiens (REF)	upload_1 (▽ Hierarchy NEW! ⓘ)					
GO biological process complete	#	#	expected	Fold Enrichment	+/-	raw P value	FDR
angiogenesis	318	11	1.53	7.20	+	5.04E-07	7.96E-03
↳anatomical structure formation involved in morphogenesis	883	16	4.24	3.77	+	5.41E-06	1.07E-02
↳anatomical structure morphogenesis	2158	26	10.37	2.51	+	8.38E-06	1.32E-02
↳blood vessel morphogenesis	410	11	1.97	5.58	+	5.49E-06	9.64E-03
↳tube morphogenesis	654	14	3.14	4.45	+	3.52E-06	1.11E-02
↳tube development	856	16	4.11	3.89	+	3.67E-06	9.67E-03
↳blood vessel development	492	13	2.37	5.50	+	8.53E-07	6.74E-03
↳vasculature development	514	13	2.47	5.26	+	1.37E-06	7.22E-03
↳circulatory system development	877	16	4.22	3.80	+	4.97E-06	1.12E-02
regulation of angiogenesis	282	9	1.36	6.64	+	1.09E-05	1.56E-02
↳regulation of anatomical structure morphogenesis	967	16	4.65	3.44	+	1.65E-05	2.00E-02
↳regulation of developmental process	2461	28	11.83	2.37	+	1.29E-05	1.70E-02
↳regulation of vasculature development	286	10	1.37	7.27	+	1.56E-06	6.17E-03
↳regulation of multicellular organismal development	1332	19	6.40	2.97	+	1.90E-05	2.15E-02
embryo development	991	16	4.76	3.36	+	2.21E-05	2.33E-02

Upregulated genes with FDR <0.05, max group expression value >1.

	Homo sapiens (REF)	upload_1 (▽ Hierarchy NEW! ⓘ)					
Reactome pathways	#	#	expected	Fold Enrichment	+/-	raw P value	FDR
Cholesterol biosynthesis via lathosterol	4	2	.02	85.10	+	5.00E-04	4.76E-02
↳Cholesterol biosynthesis	24	10	.14	70.92	+	3.69E-15	4.21E-12
↳Metabolism of steroids	148	15	.87	17.25	+	3.75E-14	2.14E-11
↳Metabolism of lipids	733	26	4.31	6.04	+	2.18E-13	9.94E-11
Cholesterol biosynthesis via desmosterol	4	2	.02	85.10	+	5.00E-04	4.57E-02
OAS antiviral response	9	4	.05	75.65	+	7.60E-07	1.45E-04
↳Antiviral mechanism by IFN-stimulated genes	79	9	.46	19.39	+	2.27E-09	5.76E-07
↳Interferon Signaling	196	15	1.15	13.03	+	1.67E-12	4.76E-10
↳Cytokine Signaling in Immune system	823	21	4.84	4.34	+	1.82E-08	4.15E-06
Linoleic acid (LA) metabolism	8	3	.05	63.83	+	3.10E-05	4.16E-03
↳alpha-linolenic (omega3) and linoleic (omega6) acid metabolism	12	3	.07	42.55	+	8.40E-05	8.72E-03
↳Fatty acid metabolism	174	8	1.02	7.83	+	1.18E-05	1.79E-03
Activation of gene expression by SREBF (SREBP)	40	11	.24	46.81	+	6.64E-15	5.05E-12
↳Regulation of cholesterol biosynthesis by SREBP (SREBF)	53	12	.31	38.54	+	2.80E-15	6.40E-12
alpha-linolenic acid (ALA) metabolism	12	3	.07	42.55	+	8.40E-05	9.14E-03
Interferon alpha/beta signaling	67	11	.39	27.94	+	9.50E-13	3.62E-10
Fatty acyl-CoA biosynthesis	37	5	.22	23.00	+	4.48E-06	7.30E-04
Interferon gamma signaling	91	7	.53	13.09	+	1.74E-06	3.05E-04
ISG15 antiviral mechanism	71	5	.42	11.99	+	8.30E-05	9.48E-03
PPARA activates gene expression	114	6	.67	8.96	+	7.44E-05	9.44E-03
↳Regulation of lipid metabolism by PPARalpha	115	6	.68	8.88	+	7.79E-05	9.37E-03
Metabolism of vitamins and cofactors	186	7	1.09	6.41	+	1.41E-04	1.40E-02
Unclassified	9941	30	58.41	.51	-	1.96E-07	4.06E-05

Figure S17. Gene ontology Panther enrichment analysis (http://geneontology.org/) on differently expressed genes in PNX0010 cells overexpressing PARG with FDR corrected *p* value < 0.05 and maximum group expression value > 1. Downregulated genes are enriched in angiogenesis regulation pathway, upregulated genes are enriched in cholesterol/lipids metabolism and interferon response.

Table S1. List of differently expressed genes in PNX0010 under PARG overexpression with FDR corrected *p* value >0.05, maximum gene expression value > 1 and fold change greater than 1.5.

Name	Induced vs. Control—Max Group Means	Induced vs. Control—Fold Change	Induced vs. Control—Log fold Change	Induced vs. Control—FDR *p*-value	Induced vs. Control—*p*-value
PTGDS	1.276835	−4.25774	−2.09009	0.012252	9.03×10^{-05}
SMIM11B	3.09168	−3.35737	−1.74733	0.000301	7.71×10^{-07}
AC073896.1	1.960024	−2.54514	−1.34775	0.01966	0.000173
ID1	12.5851	−2.29807	−1.20043	0	0
PARD6A	2.311063	−2.18061	−1.12473	0.016127	0.000128
COL1A1	1.251966	−2.18007	−1.12437	7.04×10^{-05}	1.48×10^{-07}
NRGN	6.116371	−1.91867	−0.9401	0.007711	4.96×10^{-05}
ID3	85.77683	−1.89658	−0.9234	0	0
ATOH8	5.430042	−1.83184	−0.87329	1.85×10^{-09}	8.61×10^{-13}
F12	2.604499	−1.74229	−0.80099	0.012049	8.65×10^{-05}
PRX	1.314763	−1.72537	−0.78691	0.00015	3.7×10^{-07}
GNAZ	1.180026	−1.67861	−0.74727	0.049763	0.000573
ADGRB2	2.462669	−1.66168	−0.73264	6.45×10^{-07}	5.11×10^{-10}
SLC44A2	4.170332	−1.66103	−0.73208	4.64×10^{-07}	3.03×10^{-10}
PGF	2.651785	−1.64697	−0.71981	0.016339	0.000131
NAPRT	5.32412	−1.62619	−0.7015	9.92×10^{-06}	1.39×10^{-08}
PRR3	3.201183	−1.60898	−0.68614	0.002764	1.31×10^{-05}
ANGPTL4	5.383392	−1.60737	−0.6847	1.03×10^{-05}	1.53×10^{-08}
SMAD6	3.640612	−1.59787	−0.67615	6.2×10^{-06}	8.08×10^{-09}
ETV4	2.235186	−1.57998	−0.65991	0.00044	1.21×10^{-06}
CDKN1C	3.685981	−1.57291	−0.65344	0.001554	6.01×10^{-06}
CDADC1	1.538472	−1.56888	−0.64973	0.037596	0.000394
IGF2_1	3.661241	−1.56377	−0.64503	5.08×10^{-06}	5.91×10^{-09}
KIFC2	6.600726	−1.54711	−0.62958	1.04×10^{-05}	1.6×10^{-08}
STMN3	5.002031	−1.53321	−0.61656	7.38×10^{-05}	1.58×10^{-07}
APLP1	2.013179	−1.5242	−0.60806	0.012102	8.74×10^{-05}
ARHGAP19	3.297472	−1.51002	−0.59457	9.08×10^{-05}	2.03×10^{-07}
LFNG	32.12265	−1.50183	−0.58672	0	0
IFI6	653.55	1.506149	0.590864	3.59×10^{-06}	3.85×10^{-09}
MYD88	10.41602	1.50862	0.593229	3.01×10^{-05}	5.06×10^{-08}
AC138894.1	4.624422	1.524475	0.608312	4.77×10^{-05}	9.11×10^{-08}
HMGCS1	12.02115	1.531706	0.615139	5.37×10^{-06}	6.51×10^{-09}
FASN	56.1791	1.534721	0.617977	8.93×10^{-07}	7.49×10^{-10}
IFITM1	159.4943	1.556607	0.638405	0.000467	1.31×10^{-06}
TNFSF13B	5.193219	1.564315	0.645531	3.13×10^{-05}	5.69×10^{-08}
CDKN2B	10.77522	1.585976	0.665371	5.2×10^{-07}	3.88×10^{-10}
DHCR7	44.88584	1.603855	0.681544	3.55×10^{-08}	1.82×10^{-11}
AL358113.1	2.246074	1.615253	0.69176	0.001805	7.82×10^{-06}
SLCO4C1	2.684362	1.618501	0.694659	4.35×10^{-06}	4.86×10^{-09}
PRUNE2	1.228858	1.643906	0.717128	0.000132	3.14×10^{-07}
OAS2	26.09501	1.699046	0.764725	1.29×10^{-10}	5.4×10^{-14}
MX2	21.20449	1.746987	0.804869	2×10^{-11}	7.44×10^{-15}
SCD	261.0701	1.794982	0.843969	0	0
INSIG1	157.7861	1.85465	0.891147	0	0
IGFBP5	1.149041	1.911475	0.934686	6.06×10^{-05}	1.24×10^{-07}
PARG	27.53599	1.945367	0.960043	0	0
SLX1B	8.134409	3.09175	1.628424	0.012029	8.57×10^{-05}

Table S2. Biological processes functional enrichment in differently expressed genes in PNX0010 cells overexpressing PARG with FDR corrected *p* value < 0.05 and maximum group expression value >1 predicted with STRING analysis for multiple proteins (https://string-db.org/). All GO terms were filtered as followed: gene count in term presented >7, strength >0.7 and FDR <0.001. Out of all GO terms three main groups were represented: regulation of lipid metabolism with focus in cholesterol biosynthesis (red), antiviral response with focus in interferon signaling (blue), and angiogenesis (green).

**term ID**	term description	observed gene count, >7	background gene count	strength, >0.7	FDR, <0.001
GO:0006695	cholesterol biosynthetic process	13	41	1.47	4.90x10^{-11}
GO:0045540	regulation of cholesterol biosynthetic process	13	42	1.46	4.90x10^{-11}
GO:0090181	regulation of cholesterol metabolic process	14	54	1.38	4.90x10^{-11}
GO:0060337	type I interferon signaling pathway	13	65	1.27	6.5x10E^{-10}
GO:0050810	regulation of steroid biosynthetic process	15	84	1.22	8.10x10^{-11}
GO:0019218	regulation of steroid metabolic process	17	112	1.15	4.90x10^{-11}
GO:0045071	negative regulation of viral genome replication	8	52	1.15	1.70x10^{-05}
GO:0008203	cholesterol metabolic process	15	109	1.1	1.20x10^{-09}
GO:0016125	sterol metabolic process	16	127	1.07	8.75x10^{-10}
GO:0045069	regulation of viral genome replication	9	85	0.99	4.60x10^{-05}
GO:0060333	interferon-gamma-mediated signaling pathway	7	69	0.97	0.00063
GO:0051607	defense response to virus	18	181	0.96	1.20x10^{-09}
GO:0046890	regulation of lipid biosynthetic process	17	174	0.96	4.50x10^{-09}
GO:0048525	negative regulation of viral process	9	93	0.95	7.95x10^{-05}
GO:0006637	acyl-CoA metabolic process	8	88	0.92	0.00038
GO:1901617	organic hydroxy compound biosynthetic process	14	161	0.9	7.28x10^{-07}
GO:0006633	fatty acid biosynthetic process	9	104	0.9	0.00017
GO:0009615	response to virus	20	270	0.83	7.14x10^{-09}
GO:0006720	isoprenoid metabolic process	9	123	0.83	0.0005
GO:0033875	ribonucleoside bisphosphate metabolic process	9	123	0.83	0.0005
GO:0034032	purine nucleoside bisphosphate metabolic process	9	123	0.83	0.0005
GO:0019216	regulation of lipid metabolic process	26	373	0.81	8.10x10^{-11}
GO:0008202	steroid metabolic process	17	248	0.8	5.21x10^{-07}
GO:0062012	regulation of small molecule metabolic process	22	332	0.79	5.83x10^{-09}
GO:0009108	coenzyme biosynthetic process	11	167	0.78	0.00017
GO:0006066	alcohol metabolic process	18	290	0.76	7.28x10^{-07}
GO:0001525	angiogenesis	17	297	0.72	4.27x10^{-06}
GO:0050792	regulation of viral process	10	174	0.72	0.00098
GO:0009165	nucleotide biosynthetic process	16	291	0.71	1.59x10^{-05}
GO:0072330	monocarboxylic acid biosynthetic process	11	200	0.71	0.00065

Table S3. List of primers used for qPCR experiments.

Primer and gene name	Sequence
Id1_F1.	GCTGTTACTCACGCCTCAA
Id1_R1	CAACTGAAGGTCCCTGATGTAG
Id2_F1	GACTCGCATCCCACTATTGTC
Id2_R1	ACTCAGAAGGGAATTCAGAAGC
Id3_F1	CGCGTCATCGACTACATTCT
Id3_R1	TCGTTGGAGATGACAAGTTCC
CSF2_F	CAGCCTCACCAAGCTCAA
CSF2_R	ATAATCTGGGTTGCACAGGAA
Serpine_F1	AGCAGCTATGGGATTCAAGATT
Serpine_R1	GGTGCTGATCTCATCCTTGTT
ANGPTL4_F	GAGGTCCTTCACAGCCTGCA
ANGPTL4_R	TTGGAGACTTTCTTCCGGTCAT
PARG_F1	AGGAAACTCACTGTTGGAGATG
PARG_R1	GGACTCGACAGCATGGTATATG
PARG_F2	CCAAAGCAGAGGACAGAAGAA
PARG_R2	AAGGTGAGGTGGAACGTATTTAG
SCD1 F1	TACCGCTGGCACATCAACTT
SCD1 R1	TTGGAGACTTTCTTCCGGTCAT
INSIG1 F1	CCTGCTGCGGGACAGC
INSIG1 R1	GTTCTCCGAGGTGACTGTCGA
hPolr2a_F1	CAACCAAGCCATTGCGCATC
Polr2a_R1	ACACCCAGCGTCACATTCTT
B2M_F1	CGCTACTCTCTCTTTCTGGC
B2M_R1	CTGGATGACGTGAGTAAACCTG

Table S4. List of differently expressed genes in PNX0010 under PARG overexpression with FDR corrected *p* value >0.05 and maximum gene expression value >1.

Name	Induced vs. Control—Max Group Means	Induced vs. Control—Fold Change	Induced vs. Control—Log Fold Change	Induced vs. Control—FDR *p*-value	Induced vs. Control—*p*-value
PTGDS	1.2768349	−4.2577356	−2.0900864	0.012252473	9.02992×10^{-05}
SMIM11B	3.09168	−3.3573683	−1.7473308	0.000300705	7.70572×10^{-07}
AC073896.1	1.9600237	−2.5451414	−1.3477458	0.019659855	0.000172545
ID1	12.585099	−2.2980748	−1.2004258	0	0
PARD6A	2.3110631	−2.1806119	−1.124733	0.016127082	0.000128488
COL1A1	1.2519664	−2.1800685	−1.1243735	7.03771×10^{-05}	1.47555×10^{-07}
NRGN	6.1163709	−1.9186661	−0.9401037	0.007710954	4.95789×10^{-05}
ID3	85.77683	−1.8965792	−0.9233996	0	0
ATOH8	5.4300423	−1.8318395	−0.8732931	1.84816×10^{-09}	8.61089×10^{-13}
F12	2.6044993	−1.7422909	−0.8009855	0.012049276	8.64552×10^{-05}
PRX	1.314763	−1.7253722	−0.7869076	0.000149968	3.70325×10^{-07}
GNAZ	1.1800258	−1.6786129	−0.7472696	0.049763291	0.000572685
ADGRB2	2.4626688	−1.6616821	−0.7326444	6.44846×10^{-07}	5.10757×10^{-10}
SLC44A2	4.1703323	−1.6610319	−0.7320798	4.6415×10^{-07}	3.02758×10^{-10}
PGF	2.651785	−1.6469672	−0.7198119	0.016339143	0.000130938
NAPRT	5.3241201	−1.6261892	−0.7014951	9.92118×10^{-06}	1.38674×10^{-08}
PRR3	3.2011831	−1.6089757	−0.6861426	0.002764208	1.31009×10^{-05}
ANGPTL4	5.3833923	−1.6073715	−0.6847034	1.02725×10^{-05}	1.53157×10^{-08}
SMAD6	3.6406119	−1.5978668	−0.6761472	6.19596×10^{-06}	8.08307×10^{-09}
ETV4	2.2351863	−1.5799805	−0.6599068	0.000440015	1.20957×10^{-06}
CDKN1C	3.6859812	−1.5729105	−0.6534366	0.001553545	6.00774×10^{-06}
CDADC1	1.5384721	−1.5688777	−0.6497329	0.037595883	0.000394124

IGF2_1	3.6612411	−1.5637709	−0.6450292	5.07583 × 10−06	5.9123 × 10−09
KIFC2	6.6007264	−1.5471111	−0.6295768	1.04106 × 10−05	1.60066 × 10−08
STMN3	5.0020312	−1.5332147	−0.6165597	7.38251 × 10−05	1.58224 × 10−07
APLP1	2.0131793	−1.5242039	−0.6080559	0.012101898	8.73966 × 10−05
ARHGAP19	3.2974718	−1.5100202	−0.5945678	9.07991 × 10−05	2.03064 × 10−07
LFNG	32.122646	−1.5018296	−0.5867211	0	0
MYE10OV	30.24067	−1.484643	−0.5701161	2.33249 × 10−05	3.69495 × 10−08
KLF2	8.6816201	−1.4806904	−0.56627	0.000330067	8.61192 × 10−07
RNF208	11.395187	−1.4771233	−0.5627902	0.000787868	2.60628 × 10−06
PXMP4	1.127322	−1.4722958	−0.5580675	0.044897746	0.00050414
RAPGEF3	1.8381585	−1.4652467	−0.5511436	0.000612097	1.88223 × 10−06
SERPINE1	63.31528	−1.4636951	−0.5496151	3.13132 × 10−05	5.6334 × 10−08
MXD3	3.8955436	−1.4620295	−0.5479725	0.001334995	4.8364 × 10−06
CCDC106	6.5866855	−1.4585538	−0.5445386	0.000852784	2.88388 × 10−06
GATA2	2.0101656	−1.457923	−0.5439145	0.004132186	2.29106 × 10−05
R3HCC1	5.2778313	−1.4517759	−0.5378188	0.035773994	0.000366691
SCN1B	2.3781562	−1.4396957	−0.525764	0.001764069	7.50817 × 10−06
POLI	1.7848536	−1.4380746	−0.5241385	0.002764208	1.31365 × 10−05
NBPF1	4.9701197	−1.4299433	−0.5159579	0.000644221	2.01103 × 10−06
RFX2	1.4123179	−1.4274362	−0.5134263	0.017716639	0.000146105
ZMYM1	3.7041585	−1.4258389	−0.511811	0.001683559	6.94049 × 10−06
RAC3	13.332164	−1.4221836	−0.5081078	0.001764069	7.39985 × 10−06
ATP6V0E102	3.4938384	−1.4175769	−0.503427	0.001615678	6.3233 × 10−06
ARHGAP45	4.8875176	−1.4173107	−0.503156	0.001287236	4.55807 × 10−06
HEY1	3.9270783	−1.4151551	−0.5009602	0.007349404	4.6227 × 10−05
MMP2	5.7719836	−1.4134266	−0.499197	0.001334995	4.85159 × 10−06
GDF15	90.156067	−1.4078665	−0.4935106	0.000415222	1.12206 × 10−06
NUDT2	9.1059779	−1.4025798	−0.4880829	0.012726326	9.48708 × 10−05
MED18	9.6028118	−1.3964581	−0.4817723	0.004554416	2.59098 × 10−05
SLCO4A1	86.544771	−1.3952363	−0.4805095	0.000612097	1.87871 × 10−06
UPP1	43.500703	−1.3942151	−0.4794532	0.000852784	2.90049 × 10−06
NDRG4	1.2212974	−1.3891881	−0.474242	0.040557655	0.000439682
LRWD1	8.9752133	−1.385862	−0.4707836	0.004074039	2.23984 × 10−05
ACBD4	4.6660307	−1.3857521	−0.4706692	0.011605952	7.99602 × 10−05
PRXL2B	14.631523	−1.3840772	−0.4689244	0.002249376	1.01658 × 10−05
BCAS4	1.7560515	−1.3828828	−0.4676789	0.012252473	9.07675 × 10−05
SWI5	7.8801276	−1.3823838	−0.4671583	0.012141742	8.88158 × 10−05
C11orf80	4.9101767	−1.3817198	−0.466465	0.013154817	0.000100517
SAMD11	4.1138483	−1.3813561	−0.4660852	0.009555789	6.27762 × 10−05
HCFC1R1	58.036551	−1.3793576	−0.4639965	0.00190244	8.50926 × 10−06
CLYBL	1.521185	−1.3773983	−0.4619458	0.018663722	0.000159133
CPT1A	6.8215074	−1.3763497	−0.4608471	0.002869063	1.39022 × 10−05
ADAM8	2.9436135	−1.3758358	−0.4603083	0.022291383	0.000201488
PMVK	25.78381	−1.3743182	−0.4587161	0.003891974	2.10348 × 10−05
TUBB3	2.5561011	−1.3725419	−0.4568502	0.025952282	0.000255134
PKIG	5.7431734	−1.3723365	−0.4566342	0.015413589	0.000122085
ECHDC3	6.4487969	−1.3712036	−0.4554428	0.018374046	0.000154094
KIF21B	1.0971491	−1.370537	−0.4547413	0.022483982	0.000204276
BAIAP3	2.0430835	−1.3697394	−0.4539014	0.024651534	0.000236603
DUSP7	1.9293368	−1.366249	−0.4502204	0.024619486	0.000235149
CIRBP	9.1495226	−1.3652002	−0.4491126	0.003546285	1.86707 × 10−05
C6orf48	139.74739	−1.3637103	−0.4475372	0.002663366	1.24091 × 10−05
GADD45A	151.05564	−1.360599	−0.444242	0.00237182	1.09402 × 10−05
ETV5	10.566256	−1.3584539	−0.4419656	2.17045 × 10−06	2.22476 × 10−09
P3H3	3.8694434	−1.35129	−0.4343373	0.040838917	0.00044537
DGCR6L	14.802396	−1.347824	−0.4306321	0.019659855	0.000172887
CSF2	80.397037	−1.347348	−0.4301225	0.006011785	3.55727 × 10−05

DHODH	4.5293432	−1.3465417	−0.4292589	0.01278058	9.70617 × 10−05
SHH	3.641638	−1.3430299	−0.4254914	0.022737729	0.000207641
CERCAM	3.7249804	−1.3425838	−0.4250122	0.018468738	0.000155749
BORCS8-MEF2B	6.524934	−1.3422244	−0.4246259	0.037845636	0.000400268
ATG16L2	2.8025101	−1.3411854	−0.4235087	0.022953005	0.000211746
SGSH	5.2527593	−1.3351016	−0.4169495	0.025477173	0.000248089
HMGN3	20.456258	−1.3350528	−0.4168968	0.022291383	0.00020058
GADD45B	54.375982	−1.3348314	−0.4166575	0.007715023	4.99645 × 10−05
RRM1	25.233437	−1.3249384	−0.4059253	6.15374 × 10−06	7.74127 × 10−09
TMEM141	104.53879	−1.3216079	−0.4022943	0.01275308	9.56644 × 10−05
ID2	15.675748	−1.3143769	−0.394379	0.025217816	0.000244388
ISY1-RAB43	7.5490906	−1.3079611	−0.3873197	0.028732105	0.000289155
MIS18A	21.629514	−1.3067631	−0.3859976	0.032142087	0.00032497
DUSP1	38.407407	−1.3035254	−0.3824187	0.022953005	0.000210952
KLC2	7.1162806	−1.3032929	−0.3821614	0.035272828	0.000358267
C1orf35	16.57029	−1.3013424	−0.3800006	0.049778025	0.000585903
CTGF	15.637387	−1.2866482	−0.3636176	0.00059336	1.74168 × 10−06
FAM220A	18.726171	−1.2256149	−0.2935057	0.011605952	8.00299 × 10−05
EPHA2	44.257539	−1.219349	−0.2861111	0.004554416	2.61004 × 10−05
RAC2	41.50162	−1.1876038	−0.2480536	0.03924035	0.000416848
GLUL	19.105368	1.18184361	0.24103914	0.044897746	0.000503905
HERPUD1	29.616749	1.19380575	0.25556811	0.025905826	0.00025347
STOM	31.923655	1.19631487	0.25859716	0.021917782	0.000196068
ARSE	39.205333	1.19852239	0.26125686	0.018941275	0.000163264
PRRC1	9.4756052	1.20296044	0.2665892	0.018361078	0.000152275
IFI44	45.95535	1.20499206	0.26902365	0.016563327	0.000134278
SOX4	15.198235	1.20556674	0.26971153	0.014808614	0.000115913
RELA	29.676263	1.20955067	0.27447121	0.010412717	6.88909 × 10−05
PCYT2	7.7009137	1.21071978	0.275865	0.020737077	0.00018454
TM4SF18	8.7748446	1.21467134	0.280566	0.023389833	0.000217955
GDA	16.239425	1.21929611	0.28604853	0.006241095	3.80927 × 10−05
ME1	27.862148	1.22186971	0.28909045	0.005848439	3.43337 × 10−05
TMEM173	17.738439	1.22308407	0.29052357	0.018374046	0.000153553
SMIM3	46.687096	1.22735294	0.29555017	0.003573375	1.89799 × 10−05
GBP1	34.39246	1.231758	0.30071885	0.002991478	1.47741 × 10−05
PRSS23	13.387782	1.23411417	0.30347586	0.003116351	1.5536 × 10−05
EPSTI1	25.419975	1.23624037	0.30595928	0.00277063	1.32961 × 10−05
PARVA	12.076202	1.24058931	0.3110256	0.001683559	6.93189 × 10−06
ACSL3	13.857028	1.2449747	0.31611642	0.001683559	6.98117 × 10−06
AKT1S1	24.076881	1.27407651	0.34945192	0.049778025	0.00058677
FADS1	35.573	1.27432295	0.34973094	0.045085084	0.000508344
STAT1	185.39399	1.27468885	0.35014513	0.042199594	0.000465979
CYTH3	20.959698	1.28187963	0.3582608	0.042735473	0.000473887
IL4I1	30.956015	1.28804972	0.36518828	0.037595883	0.0003928
FARP2	5.0415713	1.28982518	0.36717554	0.040838917	0.000447148
CYP1B1	17.958778	1.29798479	0.37627348	0.024749577	0.000238697
TGFB2	22.201368	1.29799017	0.37627946	3.13132 × 10−05	5.56891 × 10−08
THBS1	345.51764	1.29842805	0.37676607	0.018941275	0.000162426
ALDH1A1	160.73606	1.30098728	0.37960685	0.018663722	0.000158409
IFI44L	25.386166	1.30673814	0.38597006	0.017329571	0.000142105
LZTFL1	4.8227658	1.30753686	0.38685162	0.041896008	0.000460675
SAA1	605.36156	1.30886415	0.38831536	0.014094441	0.00010901
STAT2	19.512245	1.3109066	0.3905649	0.019062297	0.000165195
CYP51A1	24.473022	1.31202355	0.39179361	0.017329571	0.000141438
RABL3	10.776888	1.31218656	0.39197285	5.53143 × 10−05	1.10819 × 10−07
SLC2A3	25.140245	1.31539108	0.3954918	0.01447703	0.000112643
DHCR24	96.03053	1.31830252	0.39868148	0.01049093	7.0073 × 10−05

ACSL1	33.440425	1.3188033	0.3992294	0.011630362	8.07401×10^{-05}
ERBIN	8.930106	1.32130654	0.40196521	0.01278058	9.66267×10^{-05}
TRAM2	23.389284	1.32167487	0.40236732	0.010761124	7.27001×10^{-05}
KLHDC10	12.838222	1.32449477	0.40544215	0.011694365	8.17292×10^{-05}
CTHRC1	14.94436	1.32745704	0.40866517	0.020737077	0.000184057
BTN3A1	13.282612	1.32868249	0.40999639	0.011983256	8.48649×10^{-05}
FDFT1	25.662912	1.33286937	0.41453539	2.07443×10^{-06}	2.02968×10^{-09}
LAMA1	1.9078523	1.33425367	0.41603298	0.02428013	0.000230776
STARD4	25.797936	1.33663258	0.41860294	0.006241095	3.80844×10^{-05}
HERC6	10.580441	1.33663968	0.4186106	0.011453894	7.7914×10^{-05}
OASL	7.2486285	1.33694223	0.41893712	0.016537627	0.0001333
DEPP1	51.120715	1.33724637	0.41926528	0.006475787	3.98269×10^{-05}
KLHL4	2.9157967	1.33947501	0.42166767	0.023970952	0.000226721
SHISA9	9.6924726	1.34124525	0.42357306	0.006052968	3.60984×10^{-05}
PARP9	15.495934	1.34680391	0.42953981	0.00448326	2.52749×10^{-05}
DDX60L	4.5064475	1.34848295	0.43133728	0.009090117	5.92935×10^{-05}
EPC1	2.9422992	1.34916127	0.43206281	0.044588782	0.000496516
RORA	1.8661734	1.35077787	0.43379045	0.039767496	0.0004243
C1S	25.637673	1.35187845	0.43496544	5.20431×10^{-07}	3.8013×10^{-10}
EXT1	39.880415	1.35328778	0.43646867	0.002934887	1.43579×10^{-05}
INHBA	8.4082864	1.35710045	0.44052751	0.004461049	2.49418×10^{-05}
TRIM22	17.246668	1.35869776	0.44222456	0.003454897	1.78677×10^{-05}
PTPN13	10.475304	1.35952538	0.44310308	0.012125257	8.81303×10^{-05}
ZNF888	7.4121425	1.36165582	0.44536209	0.006241095	3.76164×10^{-05}
OGDH	22.010168	1.36261108	0.44637384	2.21367×10^{-07}	1.34081×10^{-10}
SQLE	33.370204	1.36313152	0.44692476	2.21367×10^{-07}	1.29182×10^{-10}
LIPC	2.3458898	1.36749022	0.45153051	0.023345348	0.000216453
ISG15	105.90986	1.36755144	0.4515951	0.002289688	1.04547×10^{-05}
ARHGAP29	48.633831	1.36812138	0.45219623	0.001683559	6.92173×10^{-06}
TMEM135	6.7876836	1.3691247	0.45325385	0.005155012	2.99104×10^{-05}
TRIM6	12.158944	1.37107795	0.45531059	0.003205297	1.64275×10^{-05}
DDX60	23.777613	1.37654141	0.46104802	0.001499862	5.66038×10^{-06}
PPIF	54.887499	1.37673737	0.46125338	0.001548462	5.91594×10^{-06}
SDC2	22.438613	1.37783404	0.46240212	0.001666525	6.59995×10^{-06}
PIK3IP1	5.1520918	1.38002717	0.46469667	0.01049093	7.0386×10^{-05}
MX1	104.72254	1.3821435	0.46690741	0.00096606	3.33078×10^{-06}
RHOBTB1	10.176932	1.38499506	0.46988083	0.001867691	8.26682×10^{-06}
HMGCR	10.192141	1.39089143	0.47600981	0.001480235	5.51618×10^{-06}
DAB2	13.648162	1.39365421	0.47887264	0.001038039	3.62731×10^{-06}
ACACA	16.963627	1.39396532	0.47919467	0.028666399	0.000287158
OAS1	28.506122	1.39459161	0.47984271	0.000762215	2.41488×10^{-06}
USP10	41.565511	1.39965272	0.48506891	0.000602553	1.79674×10^{-06}
ACSS2	9.4475268	1.40490097	0.49046844	0.001480235	5.51734×10^{-06}
IFIT1	11.666233	1.40793847	0.49358428	0.00076249	2.45128×10^{-06}
CCDC80	8.86878	1.41371722	0.49949357	0.000385773	1.02451×10^{-06}
LPIN1	6.099787	1.42679831	0.51278141	0.000516385	1.46762×10^{-06}
CPA4	2.6951967	1.42722881	0.51321664	0.035997713	0.000370661
FDPS	96.997493	1.42791041	0.51390547	0.000158392	3.98506×10^{-07}
LUM	4.0863633	1.43221912	0.51825223	0.003148466	1.58428×10^{-05}
OAS3	77.056061	1.4328178	0.51885516	0.003636633	1.94853×10^{-05}
VAV3	2.5695434	1.43715551	0.52321618	0.001764069	7.56159×10^{-06}
CMPK2	6.3659753	1.44169445	0.52776543	0.00059336	1.71612 × 10−06
IDI1	16.882639	1.44865437	0.53471342	0.000120123	2.79837×10^{-07}
ALDOC	16.732156	1.45791359	0.54390521	0.000112428	2.56673×10^{-07}
SAA2	52.105984	1.45919457	0.54517226	4.86642×10^{-05}	9.52288×10^{-08}
FADS2	108.94194	1.46064703	0.54660759	2.98984×10^{-05}	4.87558×10^{-08}
MSMO1	63.136926	1.46215017	0.54809149	2.72328×10^{-12}	8.88178×10^{-16}

FOLR1	4.868403	1.46334131	0.54926631	0.046265288	0.000528118
LDLR	8.6478149	1.46603869	0.55192318	7.38662×10^{-05}	1.61753×10^{-07}
ABCB1	2.1243776	1.47833981	0.56397792	0.01194582	8.40432×10^{-05}
C21orf91	5.9352848	1.49248411	0.57771558	4.67609×10^{-05}	8.7147×10^{-08}
LSS	25.053688	1.49794884	0.58298836	7.79339×10^{-06}	1.05301×10^{-08}
IFI6	653.55003	1.50614892	0.59086443	3.59487×10^{-06}	3.8523×10^{-09}
MYD88	10.41602	1.50861971	0.59322918	3.01461×10^{-05}	5.05642×10^{-08}
AC138894.1	4.6244217	1.52447495	0.60831245	4.7683×10^{-05}	9.10871×10^{-08}
HMGCS1	12.021151	1.5317055	0.61513894	5.37311×10^{-06}	6.50891×10^{-09}
FASN	56.179095	1.53472112	0.61797652	8.92672×10^{-07}	7.48642×10^{-10}
IFITM1	159.4943	1.5566074	0.63840512	0.000467312	1.30638×10^{-06}
TNFSF13B	5.1932193	1.56431482	0.64553089	3.13132×10^{-05}	5.68985×10^{-08}
CDKN2B	10.775216	1.58597614	0.66537106	5.20431×10^{-07}	3.87965×10^{-10}
DHCR7	44.885843	1.60385524	0.68154393	3.54953×10^{-08}	1.81917×10^{-11}
AL358113.1	2.2460744	1.61525253	0.69175973	0.001805238	7.82216×10^{-06}
SLCO4C1	2.6843616	1.61850139	0.69465861	4.34903×10^{-06}	4.8631×10^{-09}
PRUNE2	1.2288579	1.64390624	0.71712802	0.000132271	3.14301×10^{-07}
OAS2	26.095007	1.69904572	0.76472468	1.28675×10^{-10}	5.39568×10^{-14}
MX2	21.204493	1.74698682	0.80486873	1.99566×10^{-11}	7.43849×10^{-15}
SCD	261.07007	1.79498177	0.8439692	0	0
INSIG1	157.78608	1.85465018	0.8911471	0	0
IGFBP5	1.1490406	1.91147515	0.93468644	6.06495×10^{-05}	1.24334×10^{-07}
PARG	27.535989	1.94536738	0.96004263	0	0
SLX1B	8.134409	3.09175035	1.62842383	0.01202901	8.57494×10^{-05}
